# Neuroendocrine tumors and conotruncal cardiac defects 

**DOI:** 10.15171/jcvtr.2018.30

**Published:** 2018-09-10

**Authors:** Efrén Martínez-Quintana, Fayna Rodríguez-González

**Affiliations:** ^1^Cardiology Service. Insular-Materno Infantil University Hospital, Las Palmas de Gran Canaria, Spain; ^2^Medical and Surgical Sciences Department, Faculty of Health Sciences, University of Las Palmas de Gran Canaria, Las Palmas de Gran Canaria, Spain; ^3^Ophthalmology Service. Dr. Negrín University Hospital of Gran Canaria. Las Palmas de Gran Canaria, Spain

## Dear Editor,

In a previous article we made a brief review of pheochromocytomas (PHEO) and paragangliomas (PGL) in patients with cyanotic congenital heart disease (CHD) in relation to a case published in *Journal of Cardiovascular and Thoracic Research*.^1^ However we would like to add some remarks that we believe are of interest in relation to this infrequent association.

Catecholamine-secreting tumors that arise from chromaffin cells of the adrenal medulla and the sympathetic ganglia are referred to as PHEO and PGL, respectively. Both of them are neuroendocrine tumors arising from neural crest-derived cells or organs either in the adrenal gland or along the central sympathetic and parasympathetic chains.

Current evidence supports the idea that patients with a history of CHD and current or historical cyanosis might be at increased risk for developing PHEO-PGL. However inheritable genetic alterations^2^ have been also implicated in PHEO-PGL syndromes. Nonetheless, whether these tumors coassociate due to hypoxic stress, genetic defects or embryological alterations, or a combination of them is still a matter of debate.

People living at high altitudes are at increased risk for PHEO and PGL.^3^ Also, genetic susceptibilities resulting in the activation of hypoxia pathways within chromaffin cells have been implicated in the development of these tumors. Therefore, hypoxia in CHD patients may be a compensatory hyperplasic mechanism turning adrenal medular hyperplasia into an autonomously functioning medular tumor.^4^ However, and as reported by Opotowsky et al,^5^ although the vast majority of CHD patients in their series had a history of long-standing cyanosis many had undergone biventricular repair or had been converted to a Fontan circulation long before the diagnosis of the PHEO-PGL and were not hypoxemic at the time of diagnosis. In fact, only half of their patients were actively cyanotic and interestingly, both patients with noncyanotic CHD had aortic coarctation. For this reason hypoxemia should be an important criteria but not an exclusion criteria when relating CHD patients and PHEO-PGL.

From an embryological point of view the heart is essentially a mesoderm derivate. However, the cardiac neural crest cells are necessary for normal cardiovascular development. Several days after the neural crest cells have emigrated from the dorsal neural tube to the pharynx, a subpopulation of cells migrate into the cardiac outflow cushions. Here, they will condense to form the aorticopulmonary septation complex dividing the common arterial outflow into the aorta and pulmonary trunk.^6^ Defects in such septation may result in double outlet right ventricle, truncus arteriosus, transposition of the great arteries, overriding aorta, pulmonary hypoplasia, pulmonary atresia, aortic coarctation, interruption of aortic arch or tetralogy of Fallot most of which are cyanotic CHD. On the other hand, PHEO-PGL are highly vascularized tumors arising from neural crest-derived tissues in the chromaffin cells of the adrenal medulla or in the paraxial autonomic ganglia, respectively. These characteristics and the fact that some authors^4^ have established a strong relationship between CHD (mainly double outlet right ventricle, tetralogy of Fallot, pulmonary atresia or aortic coarctation) and PHEO-PGL may indicate an embryonic nature of this association that goes beyond hypoxemia.

As the established neural crest derivatives give rise to PHEO, extradrenal PGL, neuroblastomas and medullary thyroid carcinomas, all these tumors should be taken into account in conotruncal defects. In fact, patients with coexisting neuroblastoma and CHD suggest that abnormal migration and development of neural crest cells could be a common link between both of them. Similarly, medullary thyroid carcinomas should be ruled out as thyroid carcinoma in combination with PHEO has been reported in both familial and non-familial instances.

As systemic hypertension may be seen in CHD patients, excessive sweating, nervousness, periodic headaches, paroxysmal arrhythmias, hyperglycemia, heart failure or a change in new symptoms must give rise to the suspicion of these PHEO-PGL. More so when the prevalence of PHEO-PGL among arterial hypertensive patients in the general population ranges from 0.2%-0.6% among adults to 1.7% among children. Despite this in at least 25% of patients the tumor is discovered incidentally during computed tomography or magnetic resonance imaging of the abdomen for unrelated symptoms. However, once a pheochromocytoma is diagnosed, all patients should undergo a resection of the pheochromocytoma following appropriate medical preparation especially in the setting of congestive heart failure or decreased cardiac output findings frequently seen in some CHD patients.

## Ethical issues

Not applicable.

## Competing interests

Authors declare no conflict of interest in this study.
